# Effect of protein supplement level on the productive and reproductive parameters of replacement heifers managed in intensive grazing systems

**DOI:** 10.1371/journal.pone.0239786

**Published:** 2020-10-07

**Authors:** Andréia Ferreira Machado, Simone Elisa Facioni Guimarães, José Domingos Guimarães, Giancarlo Magalhães Santos, Alex Lopes Silva, Yame Fabres Robaina Sancler Silva, Domingos Souza Lollobrigida Netto, Pietro Vitor Felix Correa, Marcos Inácio Marcondes

**Affiliations:** 1 Department of Animal Science, Universidade Federal de Viçosa, Viçosa, Minas Gerais, Brazil; 2 Department of Veterinary Medicine, Universidade Federal de Viçosa, Viçosa, Minas Gerais, Brazil; 3 Cenva Post Graduation, Viçosa, Minas Gerais, Brazil; 4 Department of Animal Science, Universidade Federal Rural do Rio de Janeiro, Seropédica, Rio de Janeiro, Brazil; University of Florida, UNITED STATES

## Abstract

Evaluations of replacement heifers in intensively managed grazing systems in tropical conditions are warranted. Thus, we aimed to evaluate performance, muscle and mammary gland development, oocyte quality, and in vitro production of embryos of crossbred heifers grazing an intensively managed pasture and supplemented with high or low protein concentrates. Eighteen pubertal crossbred heifers (Holstein x Gyr) with an initial weight of 350 ± 8.0 kg were used in a 60-day trial. Two supplement types, 12% crude protein (CP) (S12CP) or 24% CP (S24CP), and a control treatment (mineral mixture, CON) were randomly distributed to the heifers. Throughout the experiment, four digestibility trials were performed over four consecutive days. Four ovarium pick-ups were performed to evaluate oocyte quality and in vitro embryo production. Lastly, ultrasounds of carcasses and mammary glands were performed. The intakes of dry matter (DM), digestible energy (DE), and CP were greater for supplemented (SUP) compared with CON heifers. The SUP heifers had a greater average daily gain (ADG) (645 versus 390 g/d) and rib eye area (58.78 versus 53.32 cm^2^) than the CON heifers. Oocyte recovery, quality, and follicle features were not affected by supplementation strategy. However, the cleavage rate (47.17% versus 30.31%) and blastocyst rate (27.91% versus 10.12%) were negatively affected by supplementation. The S12CP presented a blastocyst rate much lower than the S24CP (3.02% versus 17.23%). Carcass ultrasonography indicated a trend for greater rib eye area for S24CP and mammary ultrasonography indicated no effects of supplementation on mammary gland development. In summary, supplementation seems to be an appropriate strategy for satisfactory performance, with greater muscle deposition and no negative impacts on mammary gland development. However, in vitro embryo production was impaired when the animals received the supplementation with 12% CP.

## Introduction

Replacement heifers represent the future lactating cows of a dairy herd. However, as heifers rearing phase is long and expensive, an earlier age at first calving has been sought for better economic returns [1, 2]. One way to reduce the costs of replacement heifers is to use grazing systems [3]. In the tropics, feeding of dairy heifers is usually based on pasture systems [4], as increasing the proportion of forage in the diet reduces feed costs without affecting growth rates when adequate supplementation is provided [5].

An adequate supplementation program should be established for grazing heifers, as tropical grasses can rarely be considered a balanced diet [6] and can often lead to low performance [7]. In grazing systems, there is an excess of energy in the grasses during the rainy season, and protein supplementation is frequently needed [6, 8, 9]. Previous studies with beef heifers showed that supplementary nitrogen improved their performance [10, 11]. Additionally, protein supplementation improved the digestibility and performance of Holstein heifers grazing intensively managed in tropical grass (*Panicum maximum* cv. Mombaça) [12]. Besides, additional dietary protein may allow high rates of gain without excessive body fat deposition [13]. It may support greater protein synthesis in animal muscles, which reflects in a greater loin depth [12]. Additionally, an adequate nutritional management can avoid damages to the development of the mammary gland when heifers have high performance [14–16].

However, diets with a high concentration of nitrogen compounds have been associated with impaired reproductive performance of cows [17–19]. Grazing dairy cows usually ingest large amounts of rapidly rumen degradable protein RDP [20], allowing an increase in the levels of ammonia and circulating urea, which causes reproductive damage, such as retarded nuclear maturation and reduced rates of fertilization and cleavage [21], loss of the ability the granulosa cells to support oocyte maturation [22] and embryonic morphological, metabolic and genetic abnormalities [23]. Since there is a strong correlation between urea nitrogen concentration in follicular fluid and in blood [24]. Thus, oocyte quality may be influenced by the excess of urea nitrogen circulating in the bloodstream [25, 26]. Diets that generate high concentrations of plasma urea nitrogen may impair oocyte competence [21, 27, 28] and impair embryonic development [29–31].

The hypothesis of this study is that crossbred heifers (Holstein x Gyr) in intensively managed grazing systems supplemented with a high protein concentrate will have lower oocyte quality and lower embryo production than animals supplemented with a low protein concentrate, despite having similar performance. Thus, the aim of this study was to evaluate performance, nutrient intake and digestibility, muscle and mammary gland development, blood metabolic profile, oocyte quality, and in vitro production of embryos of crossbred heifers in an intensively managed grazing system supplemented with high or low protein concentrates.

## Materials and methods

All animal handling and procedures of the present study were approved by the Ethics Commission on the Use of Farm Animals of Universidade Federal de Viçosa (Viçosa, MG, Brazil), under protocol no. 022/2018. The sample size was calculated considering a power of 80% [32], 45% of difference from the experimental treatment in relation to the control treatment, and 25% of coefficient of variation [12]. The sample calculation was based on the productive parameters, based on studies from our group based on intensive grazing system with dairy heifers [12] in tropical conditions.

### Animals, experimental design, and feeding

Eighteen pubertal crossbred heifers (¾ Holstein × Gyr), with an initial weight of 350 ± 8.0 kg and age of 19 ± 1.0 months, were kept in an intensively managed rotational grazing system on *Panicum Maximum* cv Mombaça. Eighteen paddocks of 1000 m^2^ each were used, where all animals always grazed together in the same paddock. The paddock group had a rest area with 100 m^2^ of shade. The puberty of females was confirmed by the presence of the corpus luteum on gynecological examination by transrectal ultrasonography, before the beginning of the experimental period. The experimental period occurred during the rainy season, from January to April.

The animals were randomly distributed among three treatments, which were: no supplement (CON; control treatment), concentrate supplement with 12% CP (S12CP), and concentrate supplement with 24% CP (S24CP). Concentrate was offered at 0.5% of body weight (BW), while water and mineral mixture were supplied ad libitum for all heifers of all treatments. The concentrate was formulated based on corn meal and soy meal and was fed individually according to the pre-established treatment; the composition of the diet is presented in Table 1. Every day at 1100 h animals were removed from the paddocks, separated, and the supplement was individually fed. All heifers consumed 100% of the offered supplement. The amount of supplement offered to each animal was adjusted for each period according to their weight obtained from an intermediate weighing, in order to maintain the proportion of concentrate supplied at 0.5% of body weight. Animals from the CON treatment were also led to individual stalls but received no supplementation.

**Table 1:** Pasture (*Panicum maximum*, cv Mombaça) and concentrate chemical composition (DM basis).

Item, %DM otherwise stated^1^	Pasture–Period^2^				Concentrate	
	1	2	3	4	S12CP	S24CP
DM (%)	15.15	16.47	15.66	15.13	89.61	89.55
NDF	66.43	62.71	66.16	68.92	11.62	13.98
iNDF	13.34	13.77	13.56	13.21	0.89	0.92
CP	17.39	17.52	17.09	18.27	11.47	24.96
DE (Mcal/kg)	2.87	2.70	2.61	2.83	3.74	3.81
Ash	12.43	12.44	12.20	12.34	1.90	3.82

^1^DM = dry matter; NDF = neutral detergent fiber; iNDF = indigestible neutral detergent fiber; CP = crude protein; DE = Digestible energy (data estimated based on control animals).

^2^1 = February 13 to February 27, 2019; 2 = February 28 to March 14, 2019; 3 = March 15 to March 29, 2019; 4 = March 30 to April 13, 2019.

The calculations to determine the size and management of the paddocks followed the same methodology presented by [12]. Therefore, for a total of 90 d (30 d for adaptation and 60 d for the experimental period), using 18 heifers/paddock/d, 18 paddocks of 1000 m^2^ were necessary (considering a grazing efficiency of approximately 70%). The actual herbage allowance is presented in Table 2.

**Table 2:** Pasture (*Panicum maximum*, cv Mombaça) characteristics, pre and post-grazing sward height of 18 days (average days of cycle) of grazing activities.

Item^1^	Period^2^			
	1	2	3	4
Accumulated herbage (kg DM/ha/cycle)	1998.16	1387.28	2135.00	1746.02
Accumulated herbage (kg DM/paddock/cycle)	160.48	131.47	208.65	165.79
Herbage DM allowance (kg DM/animal/day)	8.91	7.30	11.59	9.21
Grazing efficiency (%)	80.69	88.32	63.27	68.30
PreGH (cm)	69.11	73.35	76.29	66.00
PostGH (cm)	36.84	36.95	37.16	32.18

^1^ Grazing efficiency *=* DMI (sum of all animals)/accumulated herbage (kg DM/paddock/cycle) × 100; PreGH = pre-grazing sward height; PostGH = post-grazing stubble height.

^2^1 = February 13 to February 27, 2019; 2 = February 28 to March 14, 2019; 3 = March 15 to March 29, 2019; 4 = March 30 to April 13, 2019.

**Fig 1:**
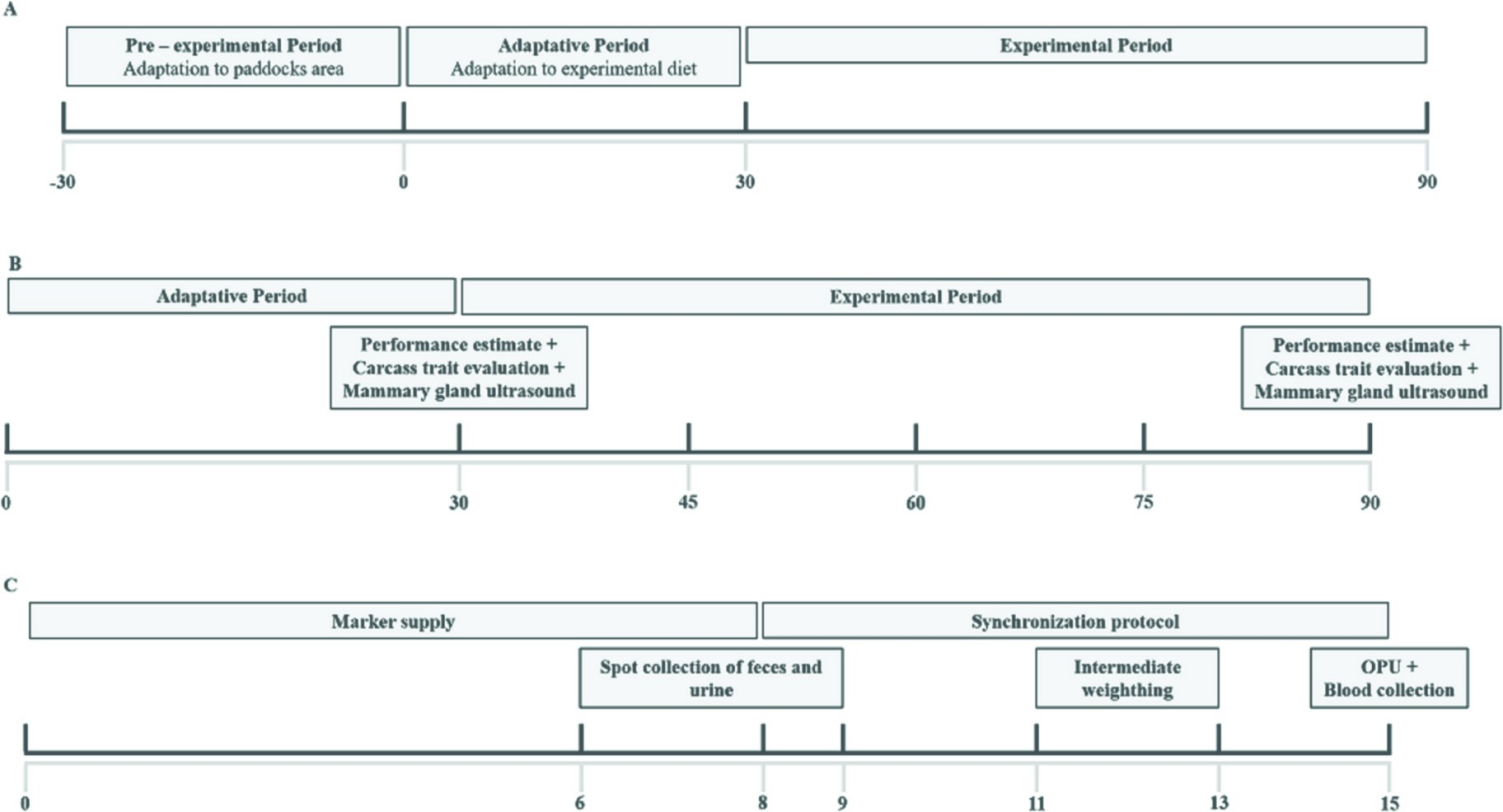
Experimental design of the different experimental phases. (A) Represents the division of the three moments of the experiment; (B) represents collections that started with the provision of experimental diets and the division of collection periods within the experimental period; (c) represents all procedures performed in each collection period (15 days).

The trial was conducted in 3 stages: pre-experimental, adaptative and experimental period. In the pre-experimental period, animals were kept in the experimental area for 30-d. During this period, all heifers were fed the same concentrate containing 18% CP at 0.5% BW. In the adaptation period, the heifers received the experimental diet for 30 d, so that the dietary effects could be observed. Lastly, the 60-d experimental period was subdivided into four periods of 15 d each. Therefore, for a period of 90 d the heifers received the experimental diet, with 30 d of adaptation and 60 d of the experimental period (Fig 1). Animals were kept in the same treatment group for the entire study.

### Performance estimates

To estimate the ADG, animals were weighed for three consecutive days at the beginning, when the experimental diet started to be provided and end of the experiment period. On the first day of each period, intermediate weightings were performed to adjust the concentrate supply. All weightings were performed after supplementation, before the animals returned to the paddock.

### Total apparent digestibility trial, analysis, and calculations

Titanium dioxide was used as a marker to determine fecal excretion, which was provided orally for eight days at 15 g/d per animal and started on d 1 of each period. After the five-day period of marker provision, fecal collection started (d 6). Four spot collections of feces were performed at 1800 h of d 6, 1400 h of d 7, 1000 h of d 8, and 0600 h of d 9 [33]. The composition and availability of the grazing stratum was estimated between d 6 and d 9 through two isolation cages (1.0 × 1.5 m), in the same way used by [12]. The forage inside the cage was cut/sampled at the same height of the pasture consumed by the animals [12, 34]. At the end of each digestibility trial, samples of pasture and feces were pooled and stored at −20 ºC.

Samples of forage and feces were partially dried in a forced-air drying oven at 55°C for 72 h [35]. Concentrate, forage, and feces samples were grounded in a Willey mill (model TE-680, brand TECNAL, Piracicaba, São Paulo, Brazil) in 2 mm and 1 mm screens [35]. The 1-mm ground samples were analyzed for DM ([36], method 934.01), CP ([36], method 990.13), ash ([36], method 942.05), and NDF corrected for ash and protein contents (NDFap) ([35], INCT-CA methods F-002/1, N-004/1, and M-002/1). While the 2-mm ground samples were used to determine the indigestible NDF, which was used as an internal marker to estimate pasture intake [37]. Feces were analyzed for titanium dioxide content ([35], M-007/1)

Four spot collections of urine were performed at 1800 h of d 6, 1400 h of d 7, 1000 h of d 8, and 0600 h of d 9, spot urine samples (approximately 50 mL) were obtained by stimulated micturition. To pool the urine samples, 10 mL of pure urine was diluted into 40 mL of sulfuric acid (0.036 *N*) and stored (−20 ºC) to prevent purine derivative degradation [38].

The urine samples were analyzed for the content of creatinine, measured using the colorimetric endpoint method (Labtest Diagnóstica S.A. Lagoa Santa, Minas Gerais, Brazil). In addition, the concentration of uric acid and allantoin in urine were determined according to Fujihara et al [39] and Chen and Gomes [40], respectively. The total daily urinary excretion was estimated using the daily creatinine excretion for Holstein heifers (CE = 32.2−0.0109×BW) [41]. The ruminal microbial CP (CPmic) synthesis was estimated as a function of absorbed purines, which was calculated from the excretion of the purine derivatives, uric acid, and allantoin, according to the equations proposed for Holstein heifers [42].

### Real-time carcass trait evaluation

On d 1 and d 90, an ultrasound device was used to measure the *gluteus medius* and the *biceps femoris* muscle intercessions and the *longissimus dorsi* [16]. We used an 18-cm linear array ultrasound instrument (Aloka SSD-500V, Aloka Co., Ltd., Tokyo, Japan) operated at a frequency of 3.5 MHz. Standoff ultrasound images (Aloka long standoff guide-beef, Aloka Co., Ltd. Tokyo, Japan) were recorded and later analyzed for back fat thickness and rib eye area using the BioSoft Toolbox® II for 200 Beef software (Biotronics Inc., Ames, Iowa, USA).

### Mammary gland ultrasound

On d 1 and d 90 we collected ultrasound images of the mammary glands. Mammary gland ultrasound images were taken using a micro-convex transducer (Mindray DP2200, Shenzhen, China), operating at a frequency of 6 MHz. Images were taken of each mammary quarter [43].

Mammary gland ultrasound images were evaluated for pixel value in 8-bit format using ImageJ^®^ software (NIH, Bethesda, MD, USA). The pixel value of each mammary quarter was obtained as the mean from three squares (16 mm^2^ each) randomly collected near the ductal structures and mammary fat pad from each image. Then, the pixel of the mammary gland was obtained as an average value of the mammary quarters.

### Blood sampling and analysis

Blood samples were collected on d 15 of each period from coccygeal venipuncture. We used vacutainer tubes with separator gel for analyses of blood urea, total protein, albumin, total cholesterol, triglycerides, insulin, and IGF-I. The tube with sodium fluoride was used for glucose analyses. The tubes were kept on ice until centrifugation (3,000 × *g* at 4 ºC for 20 min). The serum and plasma were pipetted into Eppendorf tubes and stored (−20 ºC) until analysis.

Concentrations of urea, glucose, total protein, albumin, total cholesterol, and triglycerides were measured by biochemical multi-analyzer (HumanStar 300; Human GmbH, Wesbaden, DEU). Analyses of insulin and IGF-I were performed using chemiluminescence immunoassay (Immulite 1000; Siemens Medical Solutions Diagnostics, Los Angeles, USA).

### Ultrasound-guided transvaginal follicular aspiration

On d 9 of each 15-d period, the heifers were submitted to a 6-d synchronization protocol, with 0.5 g intravaginal progesterone device (Primer–Tecnopec) insert and 2 mg estradiol benzoate (Sincrodiol, OuroFino). On d 13, the animals received 0.5 mg of cloprostenol sodium (Sincrocio, OuroFino) and the intravaginal progesterone device was removed and the ovum pick-up (OPU) occurred on d 15. An ultrasound device (B-mode) equipped with a micro-convex transducer working at a frequency of 6.5 MHz (DP2200, Mindray, China) coupled to a guide (WTA) was used. A 20 G needle and a 1.2 m follicular aspiration system (WTA, Cravinhos, SP, Brazil) were added to this system [44]. Before starting the aspiration process, the follicles present in each ovary were counted and measured. Only follicles with 8 mm or less were aspirated and the aspirated fluid was collected in a 50 mL vial containing 10 mL of 0.9% saline plus 10 IU sodium heparin/mL, and preserved at 35–36°C. All OPU procedures were standardized as described by [44].

The cumulus oocyte complexes (COC) were kept in a 0.9% saline solution to be screened, morphologically evaluated and classified as viable or inviable based on oocyte cytoplasm characteristics and the number of cumulus cell layers (adapted from [45]). Oocytes that presented layers of cumulus or were partially denuded with homogeneous cytoplasm were considered viable and oocytes nude or in degeneration that presented heterogeneous cytoplasm were considered inviable.

### In vitro oocyte maturation, fertilization, and culture

The oocytes referring to the last 3 OPUs were destined for in vitro embryo production (IVP). The oocytes were classified and those classified as viable were transferred to the maturation medium in an oocyte carrier, used to transport the oocytes to the commercial laboratory (BH Embriões, Belo Horizonte, Minas Gerais), where in vitro production was performed. After maturation, the mature COCs were fertilized with frozen sexed semen from a single batch of a single proven in vitro fertility Holstein bull. All maturation, fertilization and cultivation procedures, as well as the cleavage rate (CR) and blastocyst rate (BR), were standardized according to [44]. All media used in the procedure were purchased from Origem Embriões (Uberaba, Minas Gerais).

### Oocyte transcript quantification

The oocytes obtained at the first OPU were used for gene expression analysis. A pool of four oocytes (viables) from each female was quickly frozen in liquid nitrogen after going through the denudation process (pipetting). For total RNA extraction and cDNA synthesis, the Cells-to-cDNA kit (Ambion–Austin, USA) was used according to the manufacturer’s recommendations. Quantitation of cDNA concentration was performed using 1 μL of sample in a NanoVue Plus spectrophotometer (GE Healthcare). Finally, the samples were diluted to 10 ng/μL concentration and the material was stored at −20°C for real-time PCR analysis.

### Relative quantification by real-time PCR

Relative quantification was performed in duplicate on an ABI Prism 7300 Sequence Detection System (Applied Biosystems, Foster City, CA, USA) using GoTaq qPCR Master Mix (Promega Corporation, Madison, USA) according to the manufacturer’s recommendations. The amplification efficiency of each gene was calculated by constructing a cDNA serial dilution curve at concentrations of 25, 75, and 225 ng cDNA and concentrations of 100, 200, and 400 ng primer per reaction. The reactions were considered efficient when the amplification efficiency of the target gene and the reference gene were approximately equal, with a tolerance of 10% variation in relation to the reference gene [46]. Amplification conditions for all systems were 95°C for two minutes, 40 denaturation cycles at 95°C for 15 seconds, and extension at 60°C for 60 seconds.

The expression for each gene was calculated using the ΔCt method (target gene Ct − Ct endogenous reference) for all individual samples, where Ct reflects the PCR cycle number at which the fluorescence generated crosses an arbitrary threshold. The gene expression differences were estimated using the 2^–ΔΔCt^ method [46, 47]. Target genes evaluated in the current study were Bone Morphogenetic Protein 15 (*BMP15*) and Growth and Differentiation Factor 9 (*GDF9*) which are important regulators of ovarian follicular development and ovulation rate [48], and the reference gene was 18S ribosomal RNA (*18S*). Primer pairs for all genes are listed in S1 Table.

### Statistical analysis

All variables were analyzed using the GLIMMIX procedure of SAS (Statistical Analysis System, version 9.4). Intake and digestibility data, blood parameters, and oocyte and embryonic parameters were analyzed as a completely randomized design, and the period was included as a repeated measure in the model:

$$Y_{ijkl}=\mu +T_{i}+\delta_{ij}+P_{k}+{\left(T\;x\;P\right)}_{ik}+\varepsilon_{ijkl}$$

Where μ = general mean; T_i_ = fixed effect of the treatment i; δ_ij_ = random error with a mean of zero and variance of σ^2^, the variance among animals within treatment, equal to the covariance among repeated measures within animals; P_k_ = fixed effect of period; (T x P)_ik_ = fixed effect of the interaction between treatment i and period e; and ε_ijkl_ = random error with a mean of zero and variance of σ^2^, the variance among measures between animals.

Seven variance–covariance structures (AR1, CS, UN, TOEP, VC, ARH1, TOEPH) were tested, and the one that provided the best fit based on the Akaike information criterion was used.

Reproductive characteristics and gene expression data did not follow a normal distribution and data were analyzed using Poisson (for reproduction variables), exponential (*GDF9*), or beta (*BMP15*) distributions.

Performance data, mammary gland, carcass, and gene expression were analyzed as a completely randomized design, model:

$$Y_{ij}=\mu +T_{i}+\varepsilon_{ij}$$

Where μ = general mean; T_i_ = fixed effect of the treatment i; ε_ij_ = random error.

Initial measurements (d 0) were used as covariates in performance, mammary gland and carcass data, and were removed from the model if non-significant (P > 0.05).

Means were compared by orthogonal contrasts as follows:

Contrast 1: Effect of supplementation (non-supplemented animals–CON vs supplemented animals–SUP);

Contrast 2: levels of CP in the concentrate (S12CP vs S24CP).

Contrasts were considered significant when *P* ≤ 0.05 and tendency was used when 0.05 < *P* < 0.10.

We used data from all animals for all analyzed variables, with the exception of the gene expression analysis, which we used four animals per treatment, because not all animals produced the minimum number of viable oocytes necessary for the analysis.

## Results

The SUP had a greater dry matter intake (DMI) (*P* = 0.014; Table 3) and digestible energy (DE) intake when compared with CON (*P* < 0.01; Table 3). Additionally, the CON had a tendency to greater pasture intake (PI) and PI per BW (PI/BW) when compared with SUP (*P* = 0.05; Table 3). There was no interference of supplementation on NDF intake (4.68 kg/d), relative DMI (20.66 g/kg of BW), and relative NDF intake (12.15 g/kg of BW). The SUP had a greater CP intake when compared with CON (P = 0.008; Table 3), and there was no difference in CP digestibility between SUP and CON. Both CP intake and digestibility were greater in S24CP when compared with S12CP (*P* > 0.01; Table 3).

**Table 3:** Intake and diet digestibility of Holstein x Gyr crossbred heifers on a rotational grazing system *Panicum maximum* cv. Mombaça pasture.

Item^1^	Supplement^2^			SEM^3^	*P*-Value^4^			
	CON	S12CP	S24CP		CON × SUP	S12CP × S24CP	PER	TR × PER
Intake								
DM, kg/d	7.36	8.04	8.58	0.307	0.014	0.220	0.091	0.360
Pasture, kg/d	7.36	6.36	6.84	0.307	0.051	0.270	0.064	0.339
NDF, kg/d	4.86	4.41	4.78	0.201	0.285	0.188	0.027	0.306
CP, kg/d	1.28	1.30	1.63	0.053	0.008	0.001	0.327	0.408
CP/DM, kg/kg	0.17	0.16	0.19	0.0004	0.043	0.001	0.001	0.005
DE, Mcal/d	18.12	21.23	22.33	0.839	0.001	0.355	0.050	0.512
DM/BW, g/kg of BW	19.65	20.81	21.54	0.001	0.278	0.646	0.006	0.297
P/BW, g/kg of BW	19.65	16.49	17.23	0.001	0.056	0.641	0.004	0.301
NDF/BW, g/kg of BW	12.99	11.42	12.04	0.0007	0.176	0.553	0.002	0.259
Digestibility								
DM, g/kg	566.10	611.20	614.40	0.009	0.001	0.814	0.001	0.875
NDF, g/kg	696.10	698.20	696.60	0.005	0.864	0.844	0.001	0.893
CP, g/kg	677.30	636.70	698.60	0.015	0.608	0.010	0.001	0.472
Microbial Synthesis								
CPmic, g/d	532.10	512.40	527.74	36.954	0.794	0.772	0.924	0.284
EMS, g/kg	131.34	109.28	110.89	8.985	0.072	0.900	0.001	0.5632

^1^DM = dry matter; NDF = neutral detergent fiber; CP = crude protein; DE = Digestible energy; CP/DM = Crude protein per dry matter; DM/BW = dry matter per body weight; P/BW = pasture per body weight; NDF/BW = neutral detergent fiber per body weight; CPmic = microbial crude protein; EMS = efficiency of microbial crude protein synthesis (g of CPmic/kg of total digestible nutrients intake).

^2^CON = not supplemented; S12CP: supplemented with concentrate containing 12% CP; S24CP: supplemented with concentrate containing 24% CP.

^3^Standard error of the mean.

^4^CON × SUP = effect of supplementation; S12CP × S24CP = effect between protein levels in the supplement; PER = effect of period; TR×PER = interaction effect between supplementation and period.

The SUP animals had greater DM digestibility when compared with CON (*P* = 0.001; Table 3). The NDF digestibility was not different between SUP and CON (*P* = 0.864; Table 3). The CPmic and efficiency of microbial crude protein synthesis (EMS) were also not affected by treatment and were, on average, 524.08 g/d and 117.17 g/kg of total digestible nutrient intake, respectively.

We observed a period effect for NDF intake, relative DMI, PI, and NDF intake, DM, CP, and NDF digestibility and EMS (*P* < 0.05; Table 3). The NDF intake, relative DMI, PI, and NDF intake were greater in periods 1 and 3 when compared with periods 2 and 4. The DM, CP, and NDF digestibility were greater in period 1 when compared with other periods. However, the EMS was lower in period 1 when compared with other periods. CP intake per DMI (CPI/DMI) presented a treatment-by-period interaction, and S24CP had the greater CPI/DMI and S12CP the lowest CPI/DMI in all periods (*P* = 0.005; Table 3).

**Fig 2:**
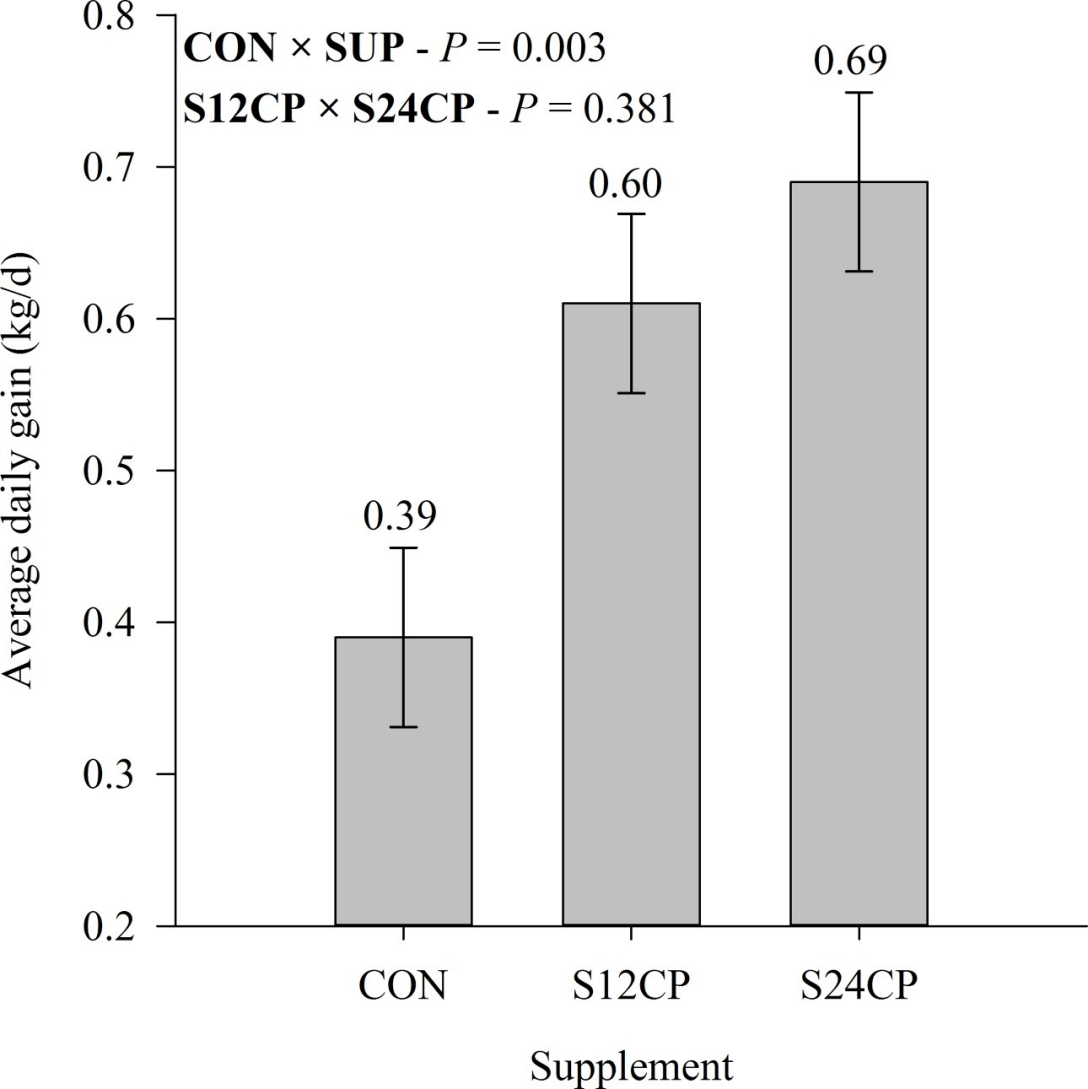
Average daily gain of Holstein x Gyr crossbred heifers on a rotational grazing system *Panicum maximum* cv. Mombaça pasture. * CON = not supplemented; S12CP: supplemented with concentrate containing 12% CP; S24CP: supplemented with concentrate containing 24% CP. *CON × SUP = effect of supplementation; S12CPB × S24CP = effect of supplement protein levels.

The ADG (*P* = 0.003; Fig 2) and rib eye area (*P* = 0.040; Table 4) were affected by supplementation, and SUP had greater ADG and rib eye area when compared with CON. We also observed a tendency for greater rib eye area in S24CP animals when compared with S12CP (*P* = 0.07; Table 4). The back fat thickness did not vary across treatments (*P* = 0.952; Table 4). The mammary glands were also not affected by treatments, either in the parenchymal tissue or in the fat pad mammary tissue (*P* > 0.05; Table 4).

**Table 4:** Carcass trait and mammary gland pixels’ pattern of Holstein × Gyr crossbred heifers on a rotational grazing system *Panicum maximum* cv. Mombaça pasture.

Item	Supplement^1^			SEM^2^	*P*-Value^3^	
	CON	S12CP	S24CP		CON × SUP	S12CP × S24CP
Rib eye area, cm^2^	53.32	56.00	61.56	1.983	0.040	0.070
Back fat thickness, mm	2.72	2.81	2.66	0.216	0.952	0.663
Parenchymal, pixels/mm^2^	4.65	4.67	4.66	0.076	0.920	0.920
Fat pad, pixels/mm^2^	5.09	5.11	5.09	0.018	0.540	0.490

^1^CON = not supplemented; S12CP: supplemented with concentrate containing 12% CP; S24CP: supplemented with concentrate containing 24% CP.

^2^Standard error of the mean.

^3^CON × SUP = effect of supplementation; S12CP × S24CP = effect between protein levels in the supplement.

**Fig 3:**
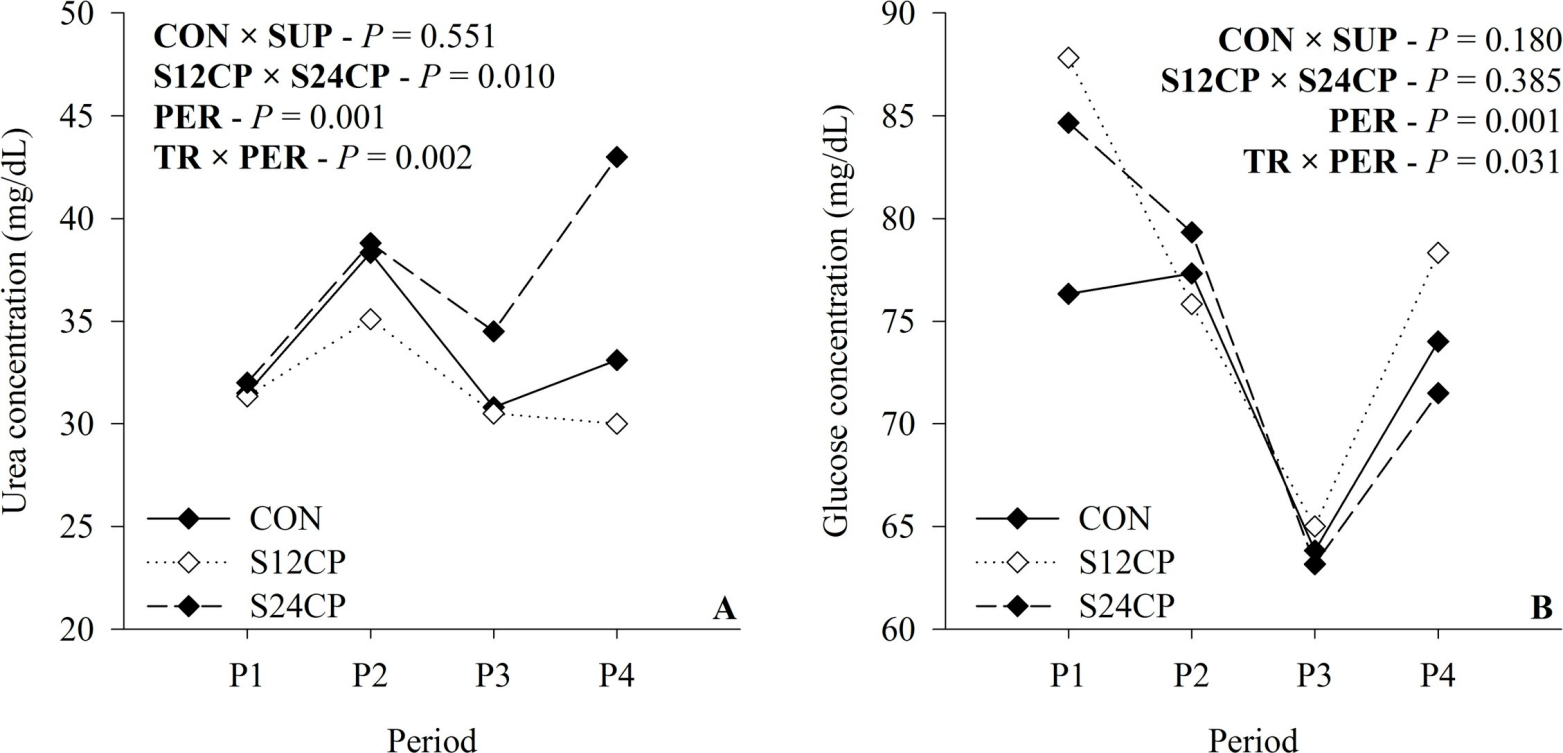
Blood urea (A) and glucose (B) concentration of Holstein x Gyr crossbred heifers on a rotational grazing system *Panicum maximum* cv. Mombaça pasture. * CON = not supplemented; S12CP: supplemented with concentrate containing 12% CP; S24CP: supplemented with concentrate containing 24% CP. *CON × SUP = effect of supplementation; S12CPB × S24CP = effect of supplement protein levels; PER = effect of period; TR×PER = interaction effect between supplementation and period.

The SUP animals had higher albumin (*P* = 0.008; Table 5) when compared with CON. Total protein (*P* = 0.873; Table 5), total cholesterol (*P* = 0.697; Table 5), triglycerides (*P* = 0.427; Table 5), insulin (*P* = 0.534; Table 5), and IGF-I (*P* = 0.959; Table 5) were not affected by treatments. Nevertheless, triglycerides, insulin, and IGF-I (*P* < 0.05; Table 5) were affected by period. Total protein had a decreased concentration in periods 3 and 4 when compared with other periods, insulin was higher during period 4 and IGF-I was lower during period 1. In addition, urea and glucose had a treatment-by-period interaction. The urea was higher in S24CP during period 4 when compared with S12CP and CON (Fig 3). The glucose was higher in S12CP during the first period when compared with CON, and, in period 4, glucose was higher in S12CP when compared with S24CP (*P* = 0.031; Fig 3).

**Table 5:** Blood parameters of Holstein x Gyr crossbred heifers on a rotational grazing system *Panicum maximum* cv. Mombaça pasture.

Item	Supplement^1^			SEM^2^	*P*-Value^3^			
	CON	S12CP	S24CP		CON × SUP	S12CP × S24CP	PER	TR × PER
Urea, mg/dL	33.45	31.75	37.08	1.286	0.551	0.010	0.001	0.002
Glucose, mg/dL	72.87	76.75	74.66	1.647	0.180	0.385	0.001	0.031
Albumin, mg/dL	2.86	3.05	3.17	0.066	0.008	0.233	0.358	0.621
Total protein, mg/dL	7.37	7.45	7.37	0.177	0.873	0.782	0.063	0.486
Total Cholesterol, mg/dL	89.62	93.91	78.87	6.656	0.697	0.130	0.941	0.850
Triglycerides, mg/dL	7.66	8.00	8.70	0.694	0.427	0.485	0.002	0.342
Insulin, μUI/mL	2.25	2.58	2.55	0.406	0.534	0.963	0.009	0.846
IGF-I, ng/mL	227.08	231.87	225.75	27.545	0.959	0.877	0.002	0.804

^1^CON = not supplemented; S12CP: supplemented with concentrate containing 12% CP; S24CP: supplemented with concentrate containing 24% CP.

^2^Standard error of the mean.

^3^CON × SUP = effect of supplementation; S12CP × S24CP = effect between protein levels in the supplement; PER = effect of period; TR×PER = interaction effect between supplementation and period

The number follicles visualized did not differ between SUP and CON (*P* = 0.273; Table 6). However, there was a difference in the follicles visualized between S24CP and S12CP, and we observed, on average, five more follicles in S24CP when compared with S12CP (*P* = 0.016; Table 6). The supplement (when compared with CON) did not change the number of oocytes recovered (*P* = 0.132; Table 6) or the recovery rate (*P* = 0.306; Table 6). Similarly, the number of viable oocytes was not affected by the CP content in the supplement (*P* = 0.926; Table 6). Additionally, the viable oocytes per oocytes recovered tended to be greater in CON animals when compared with SUP (*P* = 0.078; Table 6). The period affected the viable oocytes (*P* = 0.007; Table 6), since period 1 had the lowest viable oocytes.

**Table 6:** Reproductive parameters of Holstein x Gyr crossbred heifers on a rotational grazing system *Panicum maximum* cv. Mombaça pasture.

Item^1^	Supplement^2^			SEM^3^	*P*-Value^4^			
	CON	S12CP	S24CP		CON × SUP	S12CP × S24CP	PER	TR × PER
Follicles visualized, no	13.31	12.83	17.89	1.100	0.273	0.016	0.992	0.998
Oocytes recovered, no	10.68	12.15	14.79	1.129	0.132	0.257	0.956	0.967
Recovery rate, %	83.92	99.31	87.95	7.395	0.306	0.298	0.919	0.066
Viable oocytes, no	6.07	5.39	7.25	1.572	0.926	0.420	0.007	0.547
Viable oocytes/Oocytes recovered, %	50.64	44.34	40.17	3.651	0.078	0.398	0.081	0.090
Cleaved Oocytes, no	2.81	1.53	2.51	0.406	0.099	0.082	0.666	0.528
CR, %	47.17	23.92	36.71	5.716	0.017	0.102	0.676	0.359
IVPE, no	1.66	0.27	1.33	0.281	0.016	0.011	0.845	0.721
Blastocysts/Cleaved oocytes, %	58.30	15.26	44.57	9.436	0.019	0.055	0.214	0.666

^1^CR = cleavage rate; IVPE = in vitro produced embryos.

^2^CON = not supplemented; S12CP: supplemented with concentrate containing 12% CP; S24CP: supplemented with concentrate containing 24% CP.

^3^Standard error of the mean.

^4^CON × SUP = effect of supplementation; S12CP × S24CP = effect between protein levels in the supplement; PER = effect of period; TR×PER = interaction effect between supplementation and period

The cleaved oocytes tended to be greater in CON when compared with SUP (*P* = 0.099; Table 6). Moreover, S24CP tended to have greater cleaved oocytes when compared with S12CP (*P* = 0.082; Table 6). The CR was lower in SUP when compared with CON (*P* = 0.017; Table 6). Additionally, the CR was not different between S24CP and S12CP (*P* = 0.102; Table 6).

**Fig 4:**
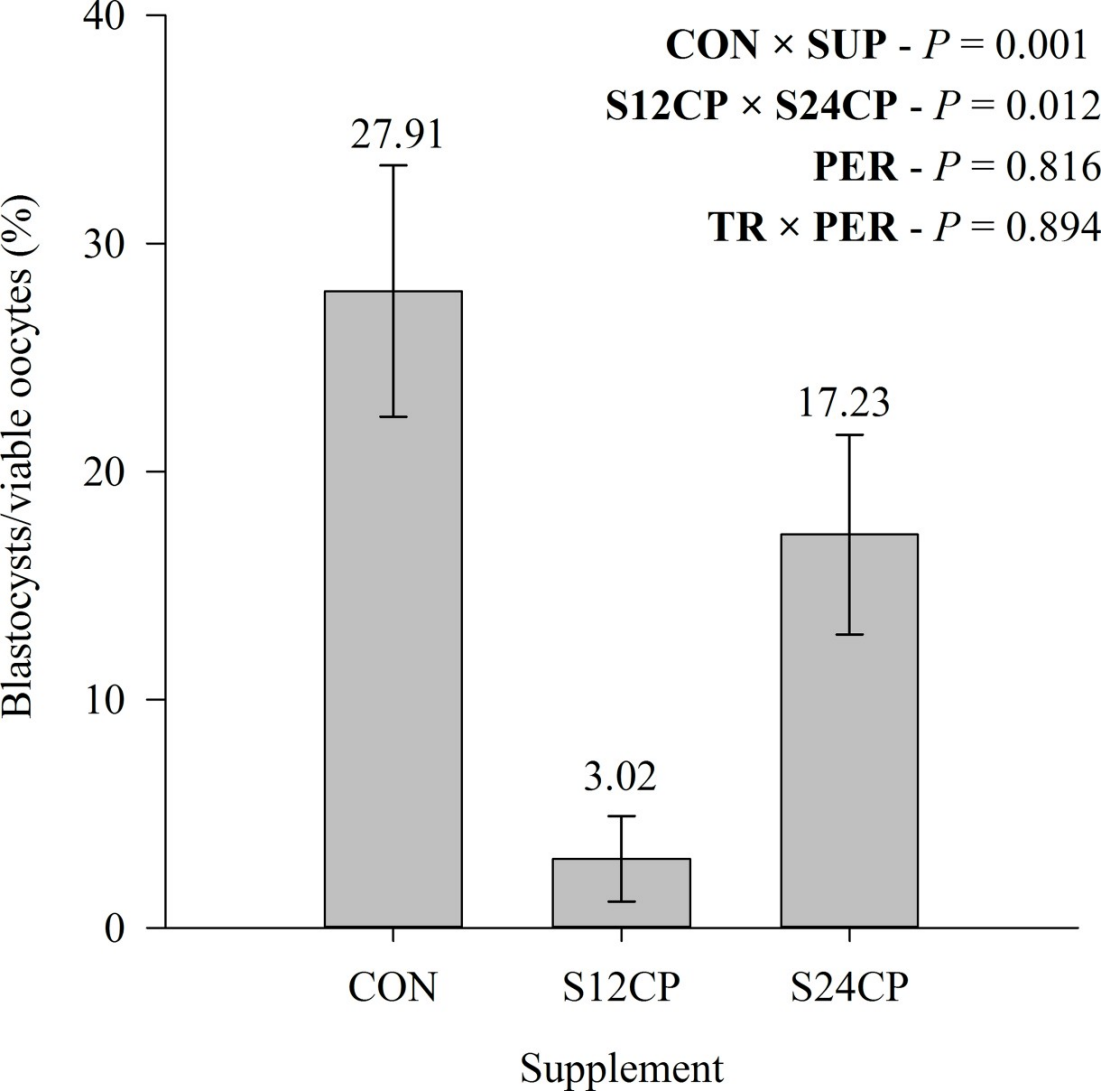
Blastocyst rate of Holstein x Gyr crossbred heifers on a rotational grazing system *Panicum maximum* cv. Mombaça pasture. * CON = not supplemented; S12CP: supplemented with concentrate containing 12% CP; S24CP: supplemented with concentrate containing 24% CP. *CON × SUP = effect of supplementation; S12CP × S24CP = effect of supplement protein levels; PER = effect of period; TR×PER = interaction effect between supplementation and period.

The supplementation negatively interfered with the BR, which was lower in SUP animals (*P* = 0.001; Fig 4). The S24CP had a greater BR when compared with S12CP (*P* = 0.012; Fig 4). The number of in vitro produced embryos (IVPE) was also negatively affected by supplementation (*P* = 0.016; Table 6). In addition, S24CP presented a greater IVPE when compared with S12CP (*P* = 0.011; Table 6). The CON had a high blastocysts per cleaved oocytes when compared with SUP (*P* = 0.019; Table 6). In addition, S24CP presented a tendency to greater blastocysts per cleaved oocytes when compared with S12CP (*P* = 0.055; Table 6). The blastocysts per cleaved oocytes averages were 58.30%, 15.26%, and 44.57% for CON, S12CP, and S24CP, respectively. Neither of these characteristics had a treatment-by-period interaction (*P* > 0.05; Table 6).

**Fig 5:**
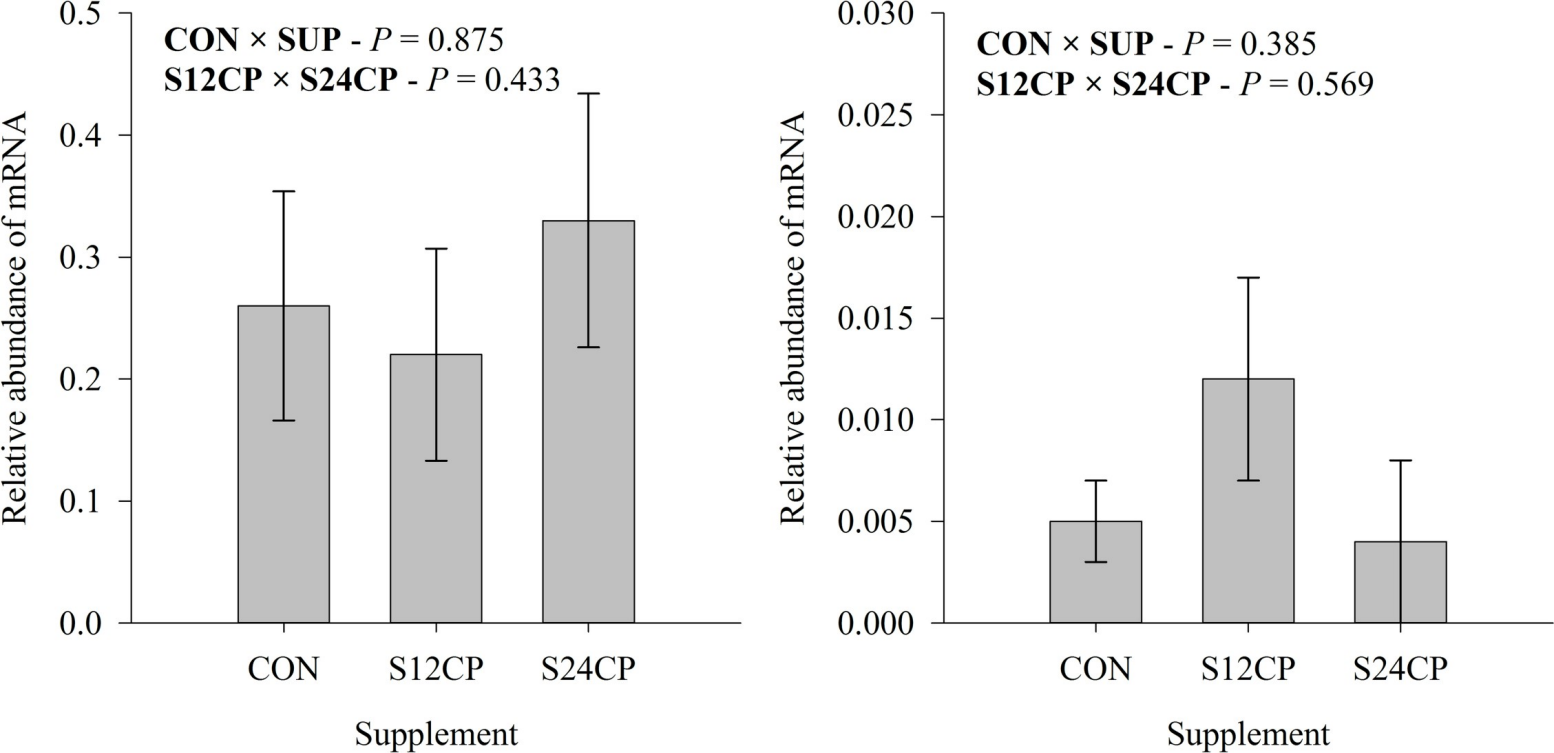
Relative abundance of A) BMP15 and B) GDF9 obtained by real-time PCR of oocytes of Holstein x Gyr crossbred heifers on a rotational grazing system *Panicum maximum* cv. Mombaça pasture. * CON = not supplemented; S12CP: supplemented with concentrate containing 12% CP; S24CP: supplemented with concentrate containing 24% CP. *CON × SUP = effect of supplementation; S12CP × S24CP = effect of supplement protein levels.

Finally, the gene expression of *BMP15* and *GDF9* was similar across treatments (*P* > 0.05; Fig 5).

## Discussion

We observed a high blood urea, especially during periods 2 and 4 when the pasture had a greater CP content. The high concentration of glucose, especially during the first period, was observed in the same period in which we observed a greater digestibility of DM and NDF, and greater relative PI and DM (g/kg BW), providing higher levels of circulating glucose. The difference found between CON and S12CP is possibly due to the greater intake of gluconeogenic precursors by S12CP animals. There was no difference in glucose concentration between CON and S24CP. Despite the supplementation received by S24CP animals, this was possibly due to the greater energy cost for the synthesis of urea of these animals [49] and due to the greater proportion of muscle tissue, which demands greater glucose uptake for muscle turnover. During period 3, there was a drop in circulating glucose, which coincided with the period of lower DM and NDF digestibility, which may explain this drop despite the greater availability of pasture (Table 2). The same reason given above explains the difference in glucose concentration between S24CP and S12CP during period 4.

Supplementation was associated with a greater DMI and DE intake, which was reflected in greater DM digestibility and ensured better ADG. The greater ADG associated with supplementation confirms the fact that supplementation improves performance even during periods of greater forage quality [50, 51] and that the greater energy intake is the reason for the higher performance, since performance is directly affected by energy intake [52].

The greater DMI and DM digestibility likely promoted the greater ADG and rib eye area observed in SUP when compared with CON animals. SUP animals had greater energy intake, which is closely linked with ADG. It has previously been described that greater energy intake stimulates muscle protein tissue synthesis, since protein turnover is stimulated by a higher content of energy consumed [53]. On the other hand, S24CP animals had a greater CP intake and CP digestibility, which is closely linked to rib eye area gain, thus gain composition, indicating that our protein supplementation (S24CP) favored a greater protein synthesis in animal muscle [54]. The increase in protein synthesis is usually related to greater ADG, since the energy cost to deposit protein is lower than lipid deposition [55].

Parenchymal and fat pad growth were also not affected by the treatments. Although effects on mammary gland development are more pronounced during the pre-puberty allometric growth phase [56], studies have shown that diet may have similar effects on mammary gland development among heifers of different growth stages [15, 57]. Usually, the development of the mammary gland of Holstein heifers may be impacted if different levels of metabolizable protein are fed [16]. In our case, supplemental CP levels did not influence tissue deposition in the mammary glands. Considering an ADG between 600 to 700 g/d of the supplemented groups, the absence of effect in the mammary gland development of crossbred animals is different from results observed in other studies. [58] observed that ½ Holstein × Gyr animals with an ADG of 0.9 kg/d showed impaired mammary gland development. This difference may have occurred due to the genetic merit of animals use in both studies. Using Holstein heifers, other authors did not find any impairment in mammary gland development with an ADG close to 1 kg/d [15, 16].

The follicles visualized number depends on follicular recruitment during the growth wave and improvements in nutritional condition lead to increased recruitment of small follicles [59, 60]. Metabolic hormones such as IGF-I and insulin can act to control follicular development stages independently or in synergy with gonadotropins by modulating follicular recruitment [61, 62]. For this reason, despite the difference found in follicles visualized between S24CP and S12CP, we could not associate this difference with the responses to treatments, since both groups received supplementation and we did not find variations in blood IGF-I and insulin between them. The oocytes recovered of our Holstein × Gyr crossbred heifers was close to the number of oocytes recovered from Holstein heifers [63] and Holstein cows [44, 64]. Crossbred ½ Holstein × Gyr animals had oocytes recovered pattern close to that found in Zebu animals, which was greater than that observed in this study (31.40 OR on average) [65]. Thus, we speculate that ¾ Holstein × Gyr heifers may have an ovarian physiology close to what we observe in Holstein heifers, but future studies should look more closely into this issue.

The viable oocytes was also close to that observed in Holstein heifers [63]. However, when compared with other authors, our viable oocytes was lower than observed in Holstein cows [44, 64]. Considering that the heifers were kept in a grazing system, we speculate that this lower oocyte quality might be a result of an increased heat stress (S2 Table). Nevertheless, our results were similar to those found in another study, who worked with grazing ¾ Holstein × Gyr cows [66]. Additionally, the viable oocytes per oocytes recovered we observed is in agreement with results obtained in environmental conditions of THI between 69 and 72 for crossbred Holstein × Gyr animals in grazing conditions [67], (S2 Table). Hyperthermia can directly affect follicle function, leading to changes in follicular development, dominance, steroidogenesis, and gonadotropin secretion [68], indicating that the climatic conditions of the experiment may have influenced the low oocyte quality. This may also indicate that, in conditions of heat stress, expenditures to maintain body temperature may mask any benefit from nutrition (greater protein and energy intake) [69]. Once again, we could not link viable oocytes and viable oocytes per oocytes recovered results with our nutrition responses, since differences in energy and protein intake across our treatments could not be associated with the differences we observed.

The absence of differences in *GDF9* and *BMP15* gene expression among treatments confirms that there were no differences in these genes that could interfere with embryo production. *BMP15* and *GDF9* are members of the *TGFβ* family and are expressed at all stages of bovine follicular development [70]. They are fundamental for activation of primordial follicles and for cell development and differentiation [71–73]. Authors have demonstrated that mutation in these genes triggers reproductive defects [74–76], confirming their importance for follicular development. Therefore, other genes must be studied in an attempt to seek the answer to the reduction of IVPE in SUP animals.

The IVPE and BR obtained were in agreement with information obtained for Holstein animals [44, 63]. Additionally, the greater CPI/DMI presented by treatments CON and S24CP did not affect the BR. However, only the CON presented a BR close to the average IVP found at the commercial level for *Bos taurus* breeds of 25.60% [77]. In a meta-analysis, increased dietary CP or increased CP degradability reduced the chances of conception in lactating cows [19]. Studies suggest that the deleterious effects of urea on embryo quality are probably due to deleterious changes in the follicle or oviduct [28, 29, 78]. The values of viable oocytes and gene expression had no difference across treatments for oocytes, only a trend of greater viable oocytes per oocytes recovered was observed for CON. However, considering that the smallest BR was found in S24CP compared with CON, we suspect that the higher blood urea due to the greater CP intake and digestibility led to losses in the IVPE of the S24CP, since the concentration of blood urea interferes in the composition of the follicular microenvironment [24–26]. Although S12CP had the lowest BR and IVPE, the blood urea concentration in this treatment was the lowest. On the other hand, it has been reported that serum urea concentration above 20 mg/dL could result in reproductive losses [24], thus all treatments in this experiment could present some level of reproductive loss.

Heifers raised in an intensively managed grazing system with a high protein content in the supplement showed no damage to the embryo survival rate or embryo development [79]. Additionally, an improvement in embryo quality occurred with a moderate long-term increase in protein content (18% CP) of Ayrshire heifers’ diets [80]. Other authors also found no effect of excess CP on long-term embryonic quality, suggesting that cows can adapt to a high urea content over a 10-day period, which prevents reproductive damage [81, 82]. In our study, the supplement was provided for a period of 30 days before the start of collections, what might explain the BR of the S24CP, indicating that although the BR was lower in the S24CP compared to the CON, we suspect there was an adaptive effect to the high blood urea, thus without a negative impact on BR.

We could not identify the reason for the low BR observed in S12CP animals. Three animals in this treatment did not present any oocytes that reached the blastocyst phase during the in vitro embryo production, greatly reducing the average rate in S12CP. During the experiment or in vitro embryo production, we could not observe anything that could elucidate these low results. Therefore, we do not have a physiological explanation for such a low rate, and we have never faced such a low rate in our farm conditions.

We also observed a higher albumin concentration in SUP, which may be linked to their greater DMI and DM digestibility. Usually blood albumin is related to a higher concentration of nutrients available for absorption, which requires a higher concentration of blood albumin for the transport of substances such as free fatty acids and amino acids [83]. The greater CP intake and CP digestibility of S24CP was provided by the greater CP in the supplement, consequently diluting the endogenous fraction of N, impacting CP digestibility. CPmic and EMS were not affected by treatments, what lead us to think that the excess protein of the S24PB was not used in rumen N metabolism and was exported to the bloodstream as ammonia, since the blood urea was on average 12% higher in the S24CP when compared with S12CP and CON.

In summary, grazing Holstein × Gyr heifers raised in intensively managed tropical pasture seems to need supplementation for satisfactory performance during the rainy season, as the supplementation was linked to optimized performance without negative impacts on mammary gland development. On the other hand, although supplementation did not influence oocyte quality, the supplement containing 12% of CP was associated with a low CR and BR, which highlights the importance of further research to better understand the nutrition–reproduction relationship in conditions of intensively managed pastures in tropical areas.

## Supporting information

S1 Checklist (PDF)

S1 Table Gene names, accession numbers and primers sequences. (DOCX)

S2 Table Environmental conditions throughout periods. (DOCX)

S1 Data (XLSX)
