# Supplementary material for: Effect of protein supplement level on the productive and reproductive parameters of replacement heifers managed in intensive grazing systems
Source: PLoS One. 2020 Oct 7;15(10):e0239786. doi: 10.1371/journal.pone.0239786 (PMC7540841; doi:10.1371/journal.pone.0239786)
Supplement: S1 Table — (DOCX) [file pone.0239786.s002.docx]

S1 Table: Gene names, accession numbers and primers sequences.

| Gene^1^ | Accession no.^2^ | Primer sequence^3^ |
| --- | --- | --- |
| *BMP15* | NM_001031752.1 | F: CACATACAGACCCTGGACTTTC  R: GGTGGGAATGAGTTAGGTGAAG |
| *GDF9* | NM_174681.2 | F: CCAGATGACAGAGCTTTGAG  R: GCCGAACAGTGTTGTAGAG |
| *18S* | NM_001033614 | F: CCTGCGGCTTAATTTGACTC  R: AACTAAGAACGGCCATGCAC |

^1^*BMP15* = bone morphogenetic protein 15; *GDF9* = growth and differentiation factor 9; *18S* = ribosomal RNA.

^2^Accession number in GenBank (http://www.ncbi.nlm.nih.gov).

^3^F = forward; R = reverse.
