# Supplementary material for: Effect of protein supplement level on the productive and reproductive parameters of replacement heifers managed in intensive grazing systems
Source: PLoS One. 2020 Oct 7;15(10):e0239786. doi: 10.1371/journal.pone.0239786 (PMC7540841; doi:10.1371/journal.pone.0239786)
Supplement: S2 Table — (DOCX) [file pone.0239786.s003.docx]

S2 Table: Environmental conditions throughout periods

| Item^1^ | Period^2^ | | | | |
| --- | --- | --- | --- | --- | --- |
|  | Adaptation | 1 | 2 | 3 | 4 |
| Average Temperature, °C | 24.35 | 22.94 | 23.40 | 21.37 | 22.05 |
| Minimum Temperature, °C | 18.27 | 19.53 | 19.58 | 18.15 | 18.65 |
| Maximum Temperature, °C | 32.01 | 29.73 | 31.52 | 27.94 | 28.66 |
| Rainfall, mm | 77.60 | 81.00 | 97.60 | 23.60 | 92.00 |
| Relative humidity, % | 68.64 | 82.93 | 81.86 | 83.25 | 85.30 |
| THI | 72.90 | 72.03 | 72.68 | 69.50 | 70.77 |

^1^THI = Temperature humidity index.

^2^Adaptation = January 13 to February 12, 2019; 1 = February 13 to February 27, 2019; 2 = February 28 to March 14, 2019; 3 = March 15 to March 29, 2019; 4 = march 30 to April 13, 2019.
